# AI-Driven and Automated Systems for Continuous Oxygen Saturation Monitoring in Long-Term Oxygen Therapy: Systematic Review

**DOI:** 10.2196/76506

**Published:** 2026-10-07

**Authors:** Suman Kadariya, Prajita Niraula, Bishal Poudel, Sujan Kadariya

**Affiliations:** 1 Conway Regional Medical Center, Conway, AR, United States; 2 Independent Researcher, Redcross Rd, Kathmandu, 44600, Nepal, 977 9818787009; 3 KIST Medical College, Kathmandu, Nepal; 4 Kathmandu University School of Medical Sciences, Dhulikhel, Nepal

**Keywords:** long-term oxygen therapy, artificial intelligence, machine learning, oxygen saturation monitoring, pulse oximetry, closed-loop systems, automated oxygen titration, motion artifacts, chronic obstructive pulmonary disease, systematic review

## Abstract

**Background:**

Long-term oxygen therapy (LTOT) improves outcomes in selected patients with severe chronic hypoxemia, but conventional LTOT uses fixed oxygen flow prescriptions that may not reflect changing needs during activity, sleep, or exacerbations. AI and automated oxygen systems may support continuous peripheral capillary oxygen saturation (SpO_2_) monitoring, signal-quality assessment, and adaptive oxygen titration. Evidence comparing AI-driven and non-AI automated approaches across performance, clinical readiness, LTOT applicability, and equity remains limited.

**Objective:**

This systematic review synthesized peer-reviewed evidence on AI-driven and automated systems for continuous SpO_2_ monitoring or oxygen titration relevant to adult LTOT, focusing on accuracy, motion robustness, clinical performance, demographic equity, and readiness for home or ambulatory deployment.

**Methods:**

This review followed PRISMA (Preferred Reporting Items for Systematic Reviews and Meta-Analyses) 2020 and PRISMA-S (Preferred Reporting Items for Systematic Reviews and Meta-Analyses–Search). PubMed, IEEE Xplore, Springer Link, ACM Digital Library, and supplementary MDPI publisher-level searches were searched for English-language peer-reviewed studies published during 2000 to January 2025. Eligible studies evaluated AI-driven or automated systems for continuous SpO_2_ monitoring or oxygen titration in adult LTOT-relevant populations and addressed motion artifact, low-perfusion signal management, skin tone bias, or prolonged signal stability. Two reviewers independently screened and extracted data; disagreements were resolved with a third reviewer. Risk of bias was assessed using an adapted Risk of Bias in Non-randomized Studies of Interventions (ROBINS-I) framework. Heterogeneous designs and outcomes precluded meta-analysis, so findings were synthesized narratively following Popay et al.

**Results:**

Of 928 records (926 from databases or platforms and 2 from manual reference screening), 912 remained after deduplication, 61 full texts were assessed, and 8 studies were included. Five studies evaluated AI-based systems and 3 evaluated automated non-AI oxygen delivery. AI models reported SpO_2_ estimation mean absolute error as low as 0.57% and root mean square error as low as 0.69%, but most were simulated, retrospective, or non–chronic obstructive pulmonary disease (COPD) specific. Only Cabanas et al reported skin tone–stratified bias analysis. Automated systems showed stronger clinical deployment evidence: O_2_matic maintained the target SpO_2_ 85.1% of the time versus 46.6% with manual titration, while Cirio and Nava reported mean SpO_2_ of 95% versus 93% manually. Risk of bias was moderate to serious, mainly due to participant selection, limited demographic reporting, and algorithmic transparency.

**Conclusions:**

AI-driven and automated LTOT-relevant systems address complementary gaps. AI approaches show promise for signal interpretation and personalized prediction, whereas rule-based automated systems have stronger near-term clinical evidence for oxygen titration. Evidence is limited by small study numbers, heterogeneous outcomes, limited COPD or home LTOT validation, and sparse equity reporting. Future work should prioritize longitudinal validation in diverse LTOT populations, prespecified equity outcomes, failure-mode reporting, and hybrid architectures combining AI signal intelligence with safety-critical automated control.

## Introduction

Chronic respiratory diseases, such as chronic obstructive pulmonary disease (COPD), represent a significant and growing global health burden. In 2019, COPD alone accounted for more than 3.2 million fatalities worldwide, and estimates indicate an increasing prevalence and mortality trend through 2050 [1]. Long-term oxygen therapy (LTOT) remains critical in the management of patients with severe chronic hypoxemia and has been demonstrated to improve survival, quality of life, and exercise tolerance in selected patients [2,3].

Despite its established clinical efficacy, conventional LTOT is routinely prescribed in a static and reactive modality, with oxygen flow rates determined during clinic appointments according to protocols such as the 6-minute walk test (6MWT). These fixed dosing regimens do not adequately address dynamic variations in patients’ oxygen requirements across daily activity, sleep, and acute physiological changes [4]. As a result, patients remain at risk of under-oxygenation, with the development of hypoxemia, or over-oxygenation, with the possibility of hypercapnia and oxidative injury.

The history of LTOT technology illustrates a persistent gap between technological ambition and real-world applicability. The landmark Medical Research Council (MRC) [3] and Nocturnal Oxygen Therapy Trial (NOTT) [2] trials, published in 1981 and 1980, respectively, established that supplemental oxygen delivered at fixed flow rates (typically 1‐4 L/min) improved survival in patients with chronic hypoxic cor pulmonale. The decades following these trials saw the introduction of demand-flow oxygen systems, which conserved oxygen supply by delivering pulses only during inhalation rather than continuously. While demand-flow devices reduced oxygen consumption, they continued to operate on static, preprogrammed thresholds and could not adapt to real-time changes in patient physiology. The clinical introduction of wearable pulse oximetry in the 1990s enabled continuous peripheral capillary oxygen saturation (SpO_2_) monitoring outside hospital settings but required clinician presence for interpretation and did not translate into automated titration. None of these successive generations of technology could adapt to real-time physiological variability, account for motion artifact contamination of photoplethysmographic (PPG) signals, or accommodate the well-documented demographic variation in measurement accuracy that has since been characterized in the literature [5,6].

Against this backdrop, a range of AI approaches have been applied to SpO_2_ signal processing and oxygen delivery. Gaussian process regression models have demonstrated mean absolute errors (MAEs) below 1% in SpO_2_ estimation from PPG data, with the ability to propagate uncertainty estimates alongside predictions [7,8]. Deep neural network architectures, including convolutional neural networks (CNNs), have been applied to both SpO_2_ prediction and motion artifact classification, offering the capacity to learn complex nonlinear mappings from raw waveform data [9]. Edge-AI frameworks, where inference runs locally on low-power embedded devices rather than in the cloud, have been proposed for predictive oxygen dosing that integrates historical physiological and behavioral trends [10]. Reference signal-less machine learning classifiers for PPG signal quality assessment have demonstrated the ability to flag and exclude corrupted signal segments in real time without additional hardware [11]. Despite these advances, no AI system reviewed to date has achieved sustained clinical validation in real-world, home-based LTOT populations, and the majority have been tested only in simulated or retrospective contexts.

In parallel, rule-based automated oxygen delivery systems, including the O_2_matic closed-loop device [12], the intelligent portable oxygen concentrator (iPOC) [13], and the automated titration device evaluated by Cirio and Nava [14], have undergone clinical testing in hospital and ambulatory settings. These systems dynamically adjust oxygen flow in response to continuous SpO_2_ readings within predefined thresholds, demonstrating meaningful improvements in time-in-target saturation compared to manual titration. However, these systems lack any learning component, cannot adapt to individual physiological patterns, and do not incorporate signal quality verification or demographic bias mitigation. The gap in evidence is therefore not merely technological: no systematic review has directly and explicitly compared AI-driven and non-AI automated systems across shared technical and clinical evaluation dimensions, with real-world LTOT deployment readiness and equity as primary analytical lenses.

This review addresses that gap. The following 3 research questions (RQs) guide the synthesis:

- RQ1: What is the technical performance, including SpO_2_ estimation accuracy, signal quality under motion, and demographic measurement robustness, of AI-driven systems for continuous SpO_2_ monitoring in adults with relevance to LTOT, as reported in peer-reviewed literature from 2000 to January 2025?
- RQ2: What is the clinical performance, including SpO_2_ maintenance within target ranges, usability, and patient outcomes, of automated non-AI oxygen delivery systems evaluated in LTOT-relevant clinical populations?
- RQ3: To what extent do AI-driven and automated SpO_2_ systems address equity-relevant challenges, specifically skin tone measurement bias and demographic representativeness, and demonstrate readiness for real-world home-based LTOT deployment?

## Methods

### Eligibility Criteria

This systematic review adhered to PRISMA (Preferred Reporting Items for Systematic Reviews and Meta-Analyses) 2020 reporting guidelines [15] (Checklist 1). The review was not prospectively registered, and no public protocol was prepared. Studies were eligible if they evaluated AI-driven or automated systems for continuous SpO_2_ monitoring or oxygen titration relevant to LTOT in adult populations and addressed at least one of the following LTOT-critical technical challenges: (1) motion-induced signal artifact correction, a prerequisite for ambulatory SpO_2_ monitoring given the frequency of patient movement in home-based LTOT; (2) low-perfusion or weak signal management, essential for reliable readings in patients with peripheral vascular compromise common in COPD; (3) skin tone measurement bias mitigation, given documented inaccuracies of pulse oximetry in individuals with darker skin pigmentation [5,6]; or (4) prolonged signal stability (>24 h), required for the continuous monitoring that defines LTOT. Technical validation outcomes such as MAE (<2%) or root mean square error (RMSE; <3%) were prioritized, as were demographic bias analyses. These criteria were designed not as arbitrary technical filters but as clinically motivated prerequisites: any SpO_2_ monitoring system intended for long-term ambulatory home use must demonstrably address each of these challenges to be considered viable for the LTOT context.

Studies were excluded if they were limited to acute or nonchronic conditions (eg, surgical hypoxia, asthma exacerbations), described interventions not relevant to LTOT (eg, nonautomated pulse rate estimation), were non–peer-reviewed (preprints, theses, conference abstracts), were inaccessible due to paywall restrictions, were published in languages other than English, or lacked explicit relevance to LTOT monitoring (eg, AI applied to electrocardiogram [ECG] analysis).

### Information Sources and Search Strategy

A systematic literature search was conducted in January 2025 across PubMed, IEEE Xplore, Springer Link, ACM Digital Library, and supplementary MDPI publisher-level searches. The search covered publications from January 1, 2000, to January 2025. The search strategy combined controlled vocabulary and free-text terms using Boolean operators to maximize sensitivity. Search terms were organized around three domains: (1) clinical context terms addressing RQ1 and RQ2, including “long-term oxygen therapy,” “LTOT,” “oxygen concentrator,” “oxygen titration,” and “SpO_2_ monitoring”; (2) technology terms addressing RQ1 and RQ2, including “artificial intelligence,” “machine learning,” “automated oxygen delivery,” “closed-loop control,” and “predictive modeling”; and (3) signal quality terms addressing RQ1 and RQ3, including “motion artifact correction,” “photoplethysmography,” “PPG,” “skin tone bias,” and “signal quality.” Complete search strings with all Boolean operators, field tags, date ranges, language filters, and records retrieved per source are documented in Multimedia Appendix 1, structured in accordance with the PRISMA-S (Preferred Reporting Items for Systematic Reviews and Meta-Analyses–Search) reporting guidelines [16] (Checklist 2

MDPI and Springer Link were searched as supplementary publisher or platform sources, not as formal bibliographic databases, to improve coverage of open-access engineering and biomedical sensor literature. Due to platform limitations, MDPI was searched using discrete keyword combinations; each string is listed separately in Multimedia Appendix 1. Because publisher-platform searching can introduce coverage bias, these sources were treated as supplementary and this limitation is acknowledged in the *Discussion* section.

### Study Selection Process

Titles and abstracts were screened independently by 2 reviewers (PN and SK) against the predefined eligibility criteria. Full-text articles meeting the criteria at the abstract stage were retrieved and independently assessed. Disagreements at both stages were resolved by consensus discussion with a third reviewer (BP). The study selection process is documented in accordance with the PRISMA 2020 guidance [15].

### Data Extraction

Data were extracted independently by 2 reviewers (PN and SK) using a prespecified extraction form developed a priori. Extracted items included the following: study design and setting, population characteristics (sample size, clinical or simulated cohort, patient diagnoses, age range where reported, and sex where reported), technology type and algorithm or device description, primary outcomes and reported performance metrics (MAE, RMSE, time-in-target SpO_2_, mean SpO_2_, functional outcomes), signal quality and bias-related findings, LTOT applicability indicators (deployment context, real-world, or simulated validation), and risk of bias domain ratings. Disagreements on extracted values were resolved through discussion with the third reviewer (BP). The extraction form template is provided in Multimedia Appendix 1.

### Data Synthesis

Due to substantial heterogeneity in study designs, populations, and outcome metrics, AI studies reported MAE and RMSE from signal estimation experiments, automated system studies reported percentage time-in-target SpO_2_ from clinical trials, 1 study reported only system architecture feasibility, and quantitative meta-analysis was not feasible. The findings are therefore presented through structured qualitative synthesis following the guidance of Popay et al [17] for narrative synthesis in systematic reviews. A convergent integrated approach was applied: the findings were organized by the 3 RQs, with studies grouped by technology type and compared across common evaluation dimensions (accuracy, motion robustness, equity, real-world applicability, and clinical usability). This approach enables comparative insight across methodologically heterogeneous studies while preserving transparency about the evidence base. For evidence synthesis, studies were classified as AI-based when they used machine learning, deep learning, or statistical learning for SpO_2_ estimation, signal-quality assessment, or predictive dosing; studies were classified as automated non-AI systems when they used deterministic rule-based or threshold-driven control without a learning component.

### Risk of Bias Assessment

Risk of bias was assessed for all 8 included studies using a framework adapted from the Risk of Bias in Non-randomized Studies of Interventions (ROBINS-I). Seven domains were evaluated for each study: (1) bias due to confounding: whether unmeasured variables such as demographics, comorbidities, or dataset composition could explain reported performance; (2) bias in participant selection: whether sampling frames were representative of real-world LTOT populations, including those with varied skin tones, activity levels, and comorbidities; (3) bias in the classification of interventions: whether the technology type and intervention mechanism were transparently described; (4) bias due to deviations from intended interventions: whether performance was assessed under realistic conditions or only under controlled or ideal-case scenarios; (5) bias due to missing data: whether data loss during monitoring (eg, motion-induced dropout, sensor disconnection) was reported and addressed; (6) bias in measurement of outcomes: whether performance metrics were validated against reference standards (eg, Bland-Altman analysis against co-oximetry); and (7) bias in selection of reported results: whether failure modes, edge cases, and suboptimal performance scenarios were reported alongside peak results. Domain ratings (low, moderate, serious, critical, no information) are visualized using the robvis R package (Multimedia Appendix 2). For AI-based studies, particular attention was given to algorithmic transparency and reproducibility as forms of bias not traditionally captured in clinical risk of bias tools.

## Results

### Literature Search Results

The initial search yielded 926 records across database and supplementary publisher-platform searching, with an additional 2 articles identified through manual reference screening. Following deduplication, 912 unique records remained for title and abstract screening. Of these, 851 were excluded at the abstract stage for clearly not meeting eligibility criteria. Sixty-one full-text articles were retrieved and assessed for eligibility. Fifty-three were excluded at the full-text stage for the following reasons: focused solely on acute or surgical care settings with no LTOT applicability (n=18), lacked an AI or automation component and described passive monitoring only (n=14), not accessible in full text due to paywall restrictions (n=9), did not report SpO_2_ as a primary outcome (n=7); were in non–peer-reviewed format (conference abstract, thesis, or preprint) (n=3), and published in non-English language (n=2). These exclusion categories map directly to the eligibility criteria described above. A final total of 8 studies were included in the qualitative synthesis. The study selection process is illustrated in Figure 1.

**Figure 1.**
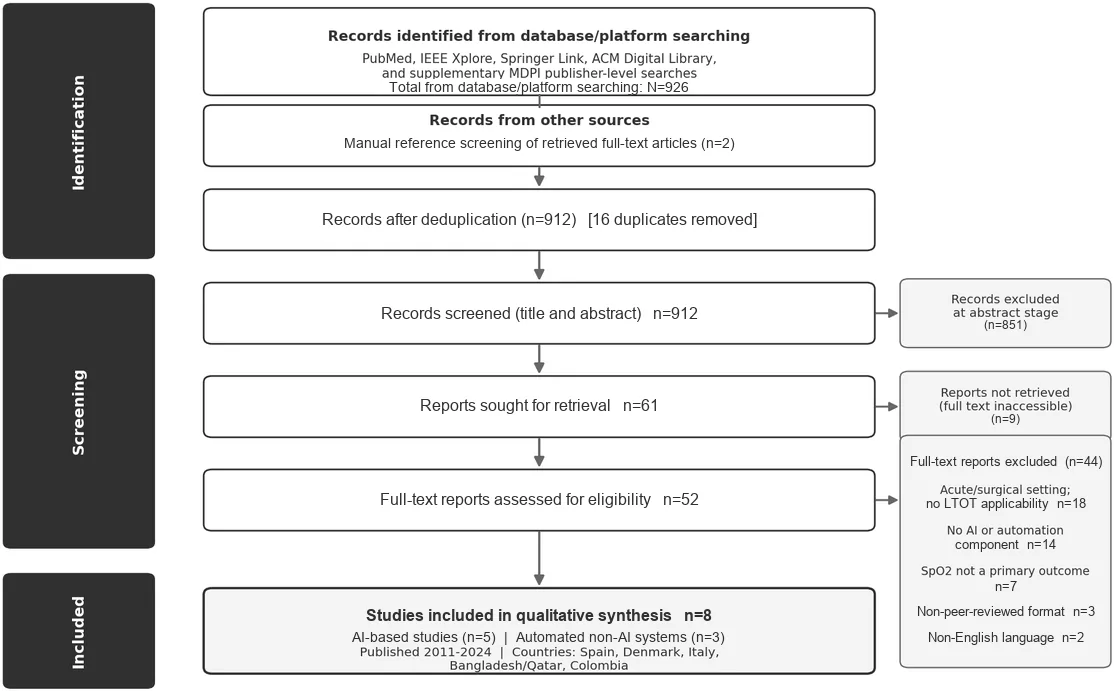
PRISMA (Preferred Reporting Items for Systematic Reviews and Meta-Analyses) 2020 flow diagram of the systematic search and study selection process. Database or platform sources included PubMed, IEEE Xplore, Springer Link, ACM Digital Library, and supplementary MDPI publisher-level searches (N=926 total), with 2 additional records identified through manual reference screening. Source-specific records reported in Multimedia Appendix 1 were PubMed (n=111), IEEE Xplore (n=23), Springer Link (n=50), ACM Digital Library (n=731), and MDPI searches (n=11). After 16 duplicates were removed, 912 records were screened at title and abstract stage. Sixty-one reports were sought for retrieval; 9 reports could not be retrieved in full text, 52 full-text reports were assessed for eligibility, and 44 full-text reports were excluded with reasons. Eight studies were included in the qualitative synthesis: 5 AI-based and 3 automated non-AI systems.

### Characteristics of the Included Studies

This systematic review included 8 studies published between 2011 and 2024, spanning 5 countries (Spain, Denmark, Bangladesh or Qatar, Italy, and Colombia). Of the 8 studies, 5 evaluated AI-based SpO_2_ monitoring systems and 3 evaluated automated non-AI oxygen delivery systems. Study designs included randomized crossover trials (n=1), pilot crossover studies (n=2), pilot usability studies (n=1), systematic and narrative reviews with model synthesis (n=2), technical validation studies (n=1), and prototype feasibility studies (n=1). Sample sizes ranged from 5 subjects in a conceptual pilot framework [10] to 19 patients in a crossover randomized controlled trial [12], with one study conducting a retrospective review of model performance across a curated multi-sensor dataset [8].

Population characteristics varied substantially between AI-based and automated system studies. The 3 automated system studies enrolled patients with COPD or chronic respiratory failure [12-14], with mean patient ages in the range of 60‐75 years where reported. In contrast, the 5 AI-based studies predominantly used healthy volunteers, retrospective PPG datasets, or simulated signal environments, without enrollment of COPD-specific populations. This represents a significant gap: AI technical validation has largely not been conducted in the populations for whom LTOT is primarily indicated. Demographic reporting was limited across all studies; only Cabanas et al [8] reported stratified analysis by skin tone, and none of the remaining 7 studies reported performance stratified by race, ethnicity, sex, or comorbidity profile (Table 1).

**Table 1.** Characteristics of included studies and key findings.

Study	Country	Study design	Population and setting	Technology type	Primary outcomes and key metrics	Real-time O_2_ titration	LTOT^a^ context	Validation type	Overall risk of bias
Cabanas et al [8], 2024	Spain	Systematic review + bias analysis	Multisensor dataset; skin tones varied; no COPD^b^-specific cohort	AI (GPR, DNN^c^)	MAE^d^: 0.57%; RMSE^e^: 0.69%; skin tone-stratified bias reported	No (simulation only)	Conceptual or simulated	Systematic review + bias analysis	Low-moderate
Shuzan et al [7], 2023	Bangladesh or Qatar	Technical validation	Healthy volunteers; simulated PPG^f^ dataset	AI (GPR, SVR^g^)	MAE: 0.57%; RMSE: 0.98% for SpO_2_^h^; also estimated respiration rate	No (simulated only)	Simulated	Technical validation	Moderate-serious
Argüello-Prada and Castillo García [11], 2024	Colombia	Narrative review + model synthesis	Not applicable (review of published ML^i^ models)	AI (ML motion detection classifiers)	Motion artifact detection performance; signal quality index analysis	N/A^j^	General framework	Narrative review	Moderate-serious
Pascual-Saldaña et al [10], 2024	Spain	Pilot framework	Five participants (diagnosis not reported); home ambulatory	AI (edge-AI, predictive dosing)	System architecture feasibility; predictive oxygen dosing concept	Yes: predictive dosing via local AI	Home LTOT (personalized)	Pilot framework	Moderate
Pascual et al [9], 2023	Spain	Prototype concept	Not reported	AI (prototype NN^k^ models)	Neural network architecture comparison; SpO_2_ prediction feasibility	No	Conceptual or simulated	Prototype concept	Serious
Sanchez-Morillo et al [13], 2020	Spain	Pilot usability study	COPD and chronic respiratory failure patients; ambulatory home setting	Automation (iPOC^l^ system)	Oxygenation stability during activity; patient satisfaction	Yes: physical activity-responsive dosing	Ambulatory or home LTOT	Pilot usability study	Moderate
Hansen et al [12], 2018	Denmark	Crossover RCT^m^ (n=19)	COPD inpatients with acute exacerbation; hospital setting	Automation (O_2_matic closed-loop)	Time-in-target SpO_2_: 85.1% (auto) vs 46.6% (manual); reduced hypoxemia time	Yes: closed-loop titration	Hospital (acute COPD)	Crossover RCT	Low-moderate
Cirio and Nava [14], 2011	Italy	Pilot crossover study (n=18)	LTOT patients during supervised exercise; hospital or rehabilitation setting	Automation (O_2_ regulator device)	Mean SpO_2_: 95% (auto) vs 93% (manual); time below target: 171 s vs 340 s	Yes: automated titration during exercise	Exercise + home LTOT transition	Pilot crossover study	Serious

a LTOT: long-term oxygen therapy.

b COPD: chronic obstructive pulmonary disease.

c DNN: deep neural network.

d MAE: mean absolute error.

e RMSE: root mean square error.

f PPG: photoplethysmography.

g SVR: support vector regression.

h SpO_2_: peripheral capillary oxygen saturation.

i ML: machine learning.

j N/A: not available.

k NN: neural network.

l iPOC: intelligent portable oxygen concentrator.

m RCT: randomized controlled trial.

### AI Versus Non-AI Classification

Included studies were classified as either AI-based or non-AI automated based on the mechanism of the primary intervention. Studies were classified as AI-based if they used machine learning, deep learning, or statistical learning models, including Gaussian process regression, support vector regression, convolutional neural networks, or feedforward neural networks, for SpO_2_ signal estimation, quality assessment, or predictive oxygen dosing. Studies were classified as non-AI automated if they used rule-based or threshold-driven closed-loop control systems that respond deterministically to real-time SpO_2_ readings without a learning component. This classification is clinically and technically meaningful: AI systems can adapt to individual patterns over time, while rule-based automated systems cannot. These distinct mechanisms produce different capability profiles and different evidence gaps, which is the central comparative insight this review seeks to establish.

### RQ1: Technical Performance of AI-Based SpO_2_ Monitoring Systems

The 5 AI-based studies reported performance using a range of metrics, reflecting the heterogeneity of their technical approaches. The 2 studies reporting direct SpO_2_ estimation accuracy achieved comparable and clinically significant results: Cabanas et al [8] reported an MAE of 0.57% and an RMSE of 0.69% using a Gaussian process regression model validated across multiple skin tones and sensor types, with Bland-Altman analysis confirming clinical-grade agreement with reference measurements. Shuzan et al [7] reported an MAE of 0.57% and an RMSE of 0.98% using machine learning models (Gaussian process regression, support vector regression) estimating SpO_2_ and respiratory rate from PPG signals, with performance assessed under controlled laboratory conditions using healthy volunteers. Both studies achieved accuracy below the commonly cited 2% MAE clinical threshold for pulse oximeter validation.

The remaining 3 AI-based studies did not report direct SpO_2_ accuracy metrics. Argüello-Prada and Castillo García [11] reviewed signal-quality-aware models capable of classifying PPG segments as artifact-contaminated or clean in real time without requiring a reference channel, a capability directly relevant to ambulatory LTOT where user movement is frequent and sensor contact variable. Pascual-Saldaña et al [10] reported system architecture feasibility for a predictive edge-AI dosing framework, not SpO_2_ accuracy per se, demonstrating viability in a 5-participant pilot but without quantified performance metrics. Pascual et al [9] compared lightweight neural network architectures for SpO_2_ prediction but lacked complete model validation or standardized error reporting, limiting the interpretation of the results.

Regarding motion artifact robustness specifically, of the 5 AI-based studies, only Argüello-Prada and Castillo García [11] directly addressed motion artifact as a primary outcome, reviewing reference signal-less classification models that flagged corrupted segments with reported accuracy. Shuzan et al [7] addressed motion robustness indirectly through feature engineering and model optimization but did not isolate motion artifact performance as a distinct outcome. Cabanas et al [8] included signal noise considerations in their bias analysis but did not report motion-specific performance metrics. The remaining 2 AI studies [9,10] did not address motion artifact handling. None of the 3 non-AI automated systems incorporated any preprocessing for motion artifact correction, operating under the assumption of continuous, valid SpO_2_ input [12-14].

### RQ2: Clinical Performance of Automated Oxygen Delivery Systems

The 3 automated system studies were evaluated using clinical efficacy metrics centered on SpO_2_ target maintenance. Hansen et al [12] conducted a randomized crossover trial (n=19) comparing the O_2_matic closed-loop system to manual oxygen titration in patients hospitalized with acute COPD exacerbation. O_2_matic maintained patients within the prescribed SpO_2_ target range 85.1% of the time versus 46.6% under manual titration, a clinically meaningful difference accompanied by significantly reduced time spent in hypoxemia. The system was described by nursing staff as safe and requiring substantially less manual intervention, suggesting a favorable clinician usability profile. Cirio and Nava [14] evaluated an automated oxygen titration device during supervised exercise in patients on LTOT (n=18), reporting mean SpO_2_ of 95% (automated) versus 93% (manual) and time below the target SpO_2_ threshold of 171 seconds versus 340 seconds under manual control. Respiratory therapist intervention time was reduced with the automated system, supporting feasibility in an outpatient exercise and rehabilitation context. Sanchez-Morillo et al [13] evaluated the iPOC system in an ambulatory home LTOT setting in patients with COPD and chronic respiratory failure, demonstrating improved oxygenation stability during physical activity and positive patient satisfaction ratings compared to conventional oxygen concentrators. Performance metrics were primarily functional rather than algorithmic.

Regarding usability across all included studies, no study applied a validated usability assessment instrument (eg, the System Usability Scale), and only 2 studies [12,13] explicitly reported clinician or patient perception of system burden and reliability. No AI-based study reported usability outcomes of any kind, consistent with their predominantly preclinical or simulation-stage nature. The absence of formal human factors evaluation across both categories of technology represents a gap in the evidence base for clinical translation.

### RQ3: Equity and Real-World LTOT Applicability

Of the 8 included studies, one explicitly addressed skin tone measurement bias: Cabanas et al [8] performed stratified analysis across skin pigmentation groups and identified measurable differences in SpO_2_ prediction error as a function of skin tone, representing the only equity-directed finding in this review. The remaining 7 studies did not report any demographic stratification by skin tone, race, ethnicity, age, or comorbidity profile. Three of the 5 AI-based studies, Shuzan et al [7], Argüello-Prada and Castillo García [11], and Pascual et al [9], relied on curated or publicly available PPG datasets; none of these datasets included demographic stratification. This finding is particularly consequential given well-documented evidence that pulse oximeters systematically overestimate SpO_2_ in individuals with darker skin pigmentation [5,6], raising the risk that AI models trained on demographically homogeneous data will propagate and amplify existing measurement inequities.

Regarding real-world LTOT deployment readiness: 2 studies demonstrated or conceptualized real-world home applicability: Sanchez-Morillo et al [13] in an ambulatory home LTOT context and Pascual-Saldaña et al [10] in a conceptual edge-AI framework for home deployment. The O_2_matic system [12] and the automated titration device by Cirio and Nava [14] were both assessed in hospital or supervised exercise environments, limiting their direct applicability to unsupervised home LTOT without further adaptation. The 3 technically strongest AI studies [7,8,11] were conducted entirely in simulated or retrospective contexts with no home LTOT deployment component. No study provided longitudinal data on system performance over the timescales relevant to LTOT, which is defined by supplemental oxygen use of 15 or more hours per day.

### Risk of Bias Assessment Results

#### Bias Due to Confounding

Of the 8 included studies, 3 AI-based studies [7,9,11] were rated at moderate risk of confounding bias, having used publicly available PPG datasets (eg, PhysioNet) that lacked full clinical context or demographic diversity. Only Cabanas et al [8] performed subgroup analysis by skin tone, identifying measurable differences in prediction error by pigmentation, reducing confounding risk in that domain. The automated system studies [12-14] were rated at moderate-to-serious confounding risk, as none stratified outcomes by comorbidities, disease severity, or socioeconomic factors.

#### Bias in Participant Selection

Shuzan et al [7] and Argüello-Prada and Castillo García [11] trained or reviewed models on precurated, high-quality PPG signals from controlled environments, raising serious risk of selection bias: these datasets overrepresent idealized sensor conditions and exclude the noisy, variable signals characteristic of real-world home-based LTOT use. Automated system studies [12,13] were tested in small, specific clinical populations, limiting generalizability to the broader, demographically diverse LTOT population. Pascual et al [9] was rated at serious selection bias risk due to prototype-only testing with no real-world population sampling.

#### Bias Due to Missing Data

Risk was generally low to moderate. Most AI studies reported complete-case analysis but did not address data loss due to motion, sensor disconnection, or prolonged monitoring interruptions. Only the iPOC system [13] explicitly described dropout detection and reinitialization protocols. No AI study conducted sensitivity analyses for missing data mechanisms (eg, distinguishing MCAR from MAR missingness), limiting the robustness of performance claims under real-world signal conditions.

#### Bias in Measurement of Outcomes

Of the AI-based studies, only Cabanas et al [8] performed Bland-Altman analysis, enabling the assessment of systematic bias and limits of agreement relative to reference measurements, the clinical standard for oximeter validation. The remaining 4 AI studies reported MAE or RMSE without reference standard validation, and Pascual et al [9] and Pascual-Saldaña et al [10] reported no quantitative SpO_2_ performance metrics at all. Among automated system studies, none validated performance against arterial blood gas (ABG) reference measurements; performance was assessed via SpO_2_-based metrics despite the acknowledged limitations of oximetry as its own reference.

#### Bias Due to Algorithmic Transparency and Reproducibility

Transparency varied substantially. Pascual-Saldaña et al [10] described model architectures and training methodology in sufficient detail to support replication. Pascual et al [9] did not provide source code, training parameters, or hyperparameter details, constituting serious reproducibility risk. Argüello-Prada and Castillo García [11] conducted a review of published models, inheriting the variable transparency of those source studies. Only Cabanas et al [8] reported demographic stratification, external validation across sensor platforms, and sufficient methodological detail to enable full reproducibility assessment.

#### Bias in Selection of Reported Results

Of the 8 included studies, only Cabanas et al [8] and Hansen et al [12] discussed performance under suboptimal or adverse conditions. Cabanas et al [8] reported bias analysis across varying skin tone groups, including cases of elevated prediction error. Hansen et al [12] reported adverse events and time spent outside the target range alongside efficacy results. The remaining 6 studies did not report failure modes, edge-case performance, or conditions under which the system underperformed, limiting the understanding of system robustness. The overall risk of bias ranged from low-moderate (Cabanas et al [8], Hansen et al [12]) to serious (Pascual et al [9], Cirio and Nava [14]), with the highest risks concentrated in participant selection, algorithmic transparency, and outcome measurement. Future studies should adopt CONSORT-AI (Consolidated Standards of Reporting Trials–Artificial Intelligence) [18] and DECIDE-AI (Developmental and Exploratory Clinical Investigations of Decision Support Systems Driven by Artificial Intelligence) [19] reporting guidelines, report failure modes transparently, and pursue model reproducibility through open-source practices (Table 2).

**Table 2.** Risk of Bias Assessment (Risk of Bias in Non-randomized Studies of Interventions [ROBINS-I] domains).

Study	Confounding	Selection	Classification of interventions	Deviations from interventions	Missing data	Outcome measurement	Reporting bias	Overall risk
Cabanas et al [8], 2024	Moderate	Moderate	Low	Low	Low	Low	Low	Low-moderate
Shuzan et al [7], 2023	Moderate	Serious	Low	Moderate	Moderate	Moderate	Moderate	Moderate-serious
Argüello-Prada and Castillo [11], 2024	Moderate	Serious	Moderate	Moderate	Moderate	Moderate	Moderate	Moderate-serious
Pascual-Saldaña et al [10], 2024	Moderate	Moderate	Low	Moderate	Moderate	Moderate	Moderate	Moderate
Pascual et al [9], 2023	Serious	Moderate	Serious	Serious	Moderate	Serious	Serious	Serious
Sanchez-Morillo et al [13], 2020	Moderate	Moderate	Low	Low	Low	Moderate	Moderate	Moderate
Hansen et al [12], 2018	Moderate	Moderate	Low	Low	Low	Moderate	Moderate	Low-moderate
Cirio and Nava [14], 2011	Serious	Moderate	Low	Low	Low	Serious	Serious	Serious

## Discussion

### Principal Findings

This systematic review synthesized findings from 8 peer-reviewed studies examining AI-driven and automated systems for continuous SpO_2_ monitoring relevant to LTOT. Across the 3 research questions, a consistent pattern emerged: AI-based systems demonstrated superior technical performance under controlled conditions, particularly in SpO_2_ estimation accuracy and signal quality management, while non-AI automated systems demonstrated superior clinical readiness and deployment evidence in real-world patient populations.

Addressing RQ1, AI models achieved SpO_2_ estimation accuracy below the 2% MAE clinical threshold in the 2 studies that reported this metric [7,8], with Cabanas et al [8] additionally providing Bland-Altman validation and skin tone–stratified bias analysis. Motion artifact handling was directly addressed only by Argüello-Prada and Castillo García [11], whose review of reference signal-less classifiers supports real-time signal quality assessment as a technically viable approach. These capabilities address specific limitations of conventional pulse oximetry in ambulatory settings. However, none of the AI-based systems reviewed have undergone real-world validation in patients with COPD or other diagnoses for which LTOT is indicated, and none have been tested over the timescales or under the environmental variability characteristic of home-based LTOT.

Addressing RQ2, the automated system evidence is more mature clinically but narrower technically. Hansen et al [12] provided the strongest evidence, demonstrating a 38.5-percentage-point improvement in time-in-target SpO_2_ over manual titration in a randomized crossover design. Sanchez-Morillo et al [13] and Cirio and Nava [14] provided supportive pilot evidence in ambulatory and exercise settings, respectively. These systems respond to current SpO_2_ readings within predefined thresholds; they cannot learn from patient history, anticipate physiological change, or adapt to signal artifact. This reactive limitation is precisely where AI-based approaches offer theoretical advantages, suggesting that hybrid systems combining closed-loop automation with AI signal intelligence and predictive modeling represent the most promising development direction.

Addressing RQ3, the equity evidence is sparse to the point of being a critical gap. Only Cabanas et al [8] performed skin tone–stratified analysis among all 8 included studies, and no study reported performance stratified by race, ethnicity, age, or comorbidity, despite the well-documented racial disparities in pulse oximetry accuracy [5,6] and the disproportionate burden of chronic respiratory disease in underrepresented populations. AI models trained predominantly on demographically homogeneous datasets risk encoding and amplifying existing measurement inequities when deployed in diverse LTOT populations. This finding aligns with and extends broader concerns in the literature about algorithmic fairness in clinical AI [20].

The maturity gap between AI and automated systems warrants explicit acknowledgment. Several AI models, including those by Pascual et al [9] and Pascual-Saldaña et al [10], remain at proof-of-concept or early prototyping stages, while O_2_matic has undergone clinical trial evaluation. This does not reflect a deficiency of AI as a technological approach, but rather the earlier stage of clinical translation for AI-based SpO_2_ systems compared to rule-based closed-loop devices.

### Strengths and Limitations

This review’s principal strengths are its explicit focus on the technical prerequisites for real-world LTOT deployment, including motion robustness, skin tone bias, and signal stability, and its comparative framing of AI-driven versus non-AI automated systems as complementary rather than competing approaches. The use of structured ROBINS-I adapted assessment, PRISMA 2020 reporting, and RQ-aligned synthesis provides a reproducible and transparent methodological foundation.

Several limitations must be acknowledged. First, the total number of included studies (n=8) is small, reflecting the novelty of the field and the scarcity of clinical trials for AI-enhanced LTOT systems; conclusions should be interpreted with this constraint in mind.

Second, restriction to English-language open-access literature may have introduced language and publication bias, potentially excluding relevant studies published in other languages or behind paywalls. Among the 9 reports that could not be retrieved in full text, article-level metadata were available for 5 records. These records appeared to address related areas including machine learning classification of COPD using pulse oximetry, smartphone-based SpO_2_ estimation, deep learning estimation of SpO_2_ from PPG signals, machine learning assessment of pulse oximeter response time, and prototype automated oxygen regulation using SpO_2_ feedback. This suggests that the inaccessible literature may have contained additional early-stage engineering, mobile-health, and device-development studies relevant to AI-enabled or automated oxygen monitoring. However, because the full texts could not be assessed, these records were not included in data extraction, risk-of-bias assessment, or synthesis. Their omission may mean that this review underrepresents low-cost prototypes, conference-proceeding technologies, and technical validation studies, but available metadata do not suggest that they would resolve the central evidence gaps identified in this review: limited real-world LTOT validation, sparse demographic equity reporting, and lack of longitudinal home-based evaluation.

Third, outcome heterogeneity prevented meta-analysis; the findings must be interpreted at the level of individual study design and setting. Fourth, this review did not systematically address regulatory approval pathways, cost-effectiveness, or implementation science considerations, all of which are necessary for translation into routine clinical practice. Fifth, this review was not prospectively registered in PROSPERO; future updates should consider prospective registration to enhance protocol transparency.

### Implications for Clinical Practice and Future Research

The convergent finding of this review, namely that AI systems and automated systems occupy complementary capability niches, has a direct implication for the clinical development agenda: hybrid architectures that pair closed-loop automated oxygen titration with AI-based signal quality assurance and predictive dosing represent the most promising near-term direction. For such systems to be clinically deployable, real-world longitudinal validation is needed in ambulatory LTOT populations that include patients across the demographic spectrum of LTOT eligibility, with prespecified equity outcomes and failure mode reporting. Edge-computing approaches [10] that enable local AI inference without cloud dependency are particularly relevant for home-based and resource-limited settings. Standardized performance reporting, including demographic stratification, motion-specific accuracy metrics, and validation against ABG or co-oximetry reference standards, should be adopted across the field to facilitate cross-study comparability and regulatory assessment.

### Conclusions

This systematic review identified 8 peer-reviewed studies addressing AI-driven and automated systems for continuous SpO_2_ monitoring in LTOT. AI-based systems demonstrated strong technical performance in SpO_2_ estimation accuracy [7,8] and signal quality management under motion [11] and offer a promising pathway to personalized, predictive oxygen therapy. Automated non-AI systems demonstrated clinical readiness, with O_2_matic producing a clinically meaningful improvement of 38.5 percentage points in time-in-target SpO_2_ compared to manual titration [12]. The central finding of this review is that these 2 technology types address different capability gaps and are best understood as complementary rather than competing approaches. Significant gaps persist across both: limited real-world validation in LTOT populations, near-total absence of demographic equity data, inconsistent outcome reporting, and inadequate algorithmic transparency. Closing these gaps through longitudinal clinical trials in diverse LTOT populations, adoption of CONSORT-AI and DECIDE-AI reporting standards, and development of hybrid AI-automated architectures will determine whether these technologies fulfill their promise of safer, more equitable, and more responsive long-term oxygen therapy.

## Supplementary material

10.2196/76506 Multimedia Appendix 1 Inclusion and exclusion criteria, search strategies, and data extraction form.

10.2196/76506 Multimedia Appendix 2 Risk of bias materials.

10.2196/76506 Checklist 1 PRISMA 2020 checklist.

10.2196/76506 Checklist 2 PRISMA-S checklist.

## Abbreviations

6MWT: 6-minute walk test
ABG: arterial blood gas
CNN: convolutional neural network
CONSORT-AI: Consolidated Standards of Reporting Trials–Artificial Intelligence
COPD: chronic obstructive pulmonary disease
DECIDE-AI: Developmental and Exploratory Clinical Investigations of Decision Support Systems Driven by Artificial Intelligence
ECG: electrocardiogram
iPOC: intelligent portable oxygen concentrator
LTOT: long-term oxygen therapy
MAE: mean absolute error
MRC: Medical Research Council
NOTT: Nocturnal Oxygen Therapy Trial
PPG: photoplethysmography
PRISMA: Preferred Reporting Items for Systematic Reviews and Meta-Analyses
PRISMA-S: Preferred Reporting Items for Systematic Reviews and Meta-Analyses–Search
RMSE: root mean square error
ROBINS-I: Risk of Bias in Non-randomized Studies of Interventions
RQ: research question
SpO_2_: peripheral capillary oxygen saturation
