# Supplementary material for: AI-Driven and Automated Systems for Continuous Oxygen Saturation Monitoring in Long-Term Oxygen Therapy: Systematic Review
Source: JMIRx Med. 2026 Oct 7;7:e76506. doi: 10.2196/76506 (PMC13646447; doi:10.2196/76506)
Supplement: Supplementary file 1 [file xmed-v7-e76506-s003.pdf]

PRISMA 2020 checklist

Checklist source: Page MJ et al. The PRISMA 2020 statement: an updated guideline for reporting systematic reviews. BMJ. 2021;372:n71. This table maps PRISMA 2020 reporting items to the revised manuscript and supplementary appendices.

Item	Topic	Location in revised submission
1	Title	Title page.
2	Abstract	Abstract.
3	Rationale	Introduction.
4	Objectives	Introduction and research questions.
5	Eligibility criteria	Methods: Eligibility Criteria; Appendix A.
6	Information sources	Methods: Information Sources and Search Strategy; Appendix A.
7	Search strategy	Appendix A.
8	Selection process	Methods: Study Selection; Results and Figure 1.
9	Data collection process	Methods: Data Extraction.
10a	Outcomes	Methods: Data Extraction; Results organized by research questions.
10b	Other variables	Methods: Data Extraction; Table 1.
11	Risk of bias assessment	Methods: Risk of Bias; Results Table 2; Appendix B.
12	Effect measures	Methods: Data Synthesis; Results report MAE, RMSE, time-in-target, and mean SpO2
13a	Synthesis eligibility	Methods: Data Synthesis.
13b	Data preparation for synthesis	Methods: Data Synthesis; no quantitative pooling was conducted.
13c	Methods for displaying results	Results Table 1, Table 2, and Figure 1.
13d	Synthesis methods	Methods: Data Synthesis; narrative synthesis based on Popay et al. guidance.
13e	Exploration of heterogeneity	Methods and Strengths and Limitations; handled narratively because studies were heterogeneous.
13f	Sensitivity analyses	Not conducted; no meta-analysis was performed.
14	Reporting bias assessment	Risk-of-bias framework, including selection of reported results domain; Appendix B.

15	Certainty assessment	No formal GRADE assessment; certainty limitations are discussed through risk-of-bias and evidence-limitations sections.
16a	Study selection results	Results and Figure 1.
16b	Excluded reports	Results full-text exclusion categories; Appendix A provides source-level screening detail.
17	Study characteristics	Results and Table 1.
18	Risk of bias in studies	Results Table 2 and Appendix B.
19	Results of individual studies	Results research-question sections and Table 1.
20a	Synthesis characteristics and risk	Results and Discussion.
20b	Statistical syntheses	Not applicable; no meta-analysis was performed.
20c	Investigations of heterogeneity	Narrative discussion only.
20d	Sensitivity analyses	Not applicable.
21	Reporting biases	Risk-of-bias reported-results domain; Appendix B.
22	Certainty of evidence	Not formally graded; limitations and risk-of-bias synthesis are reported in Discussion.
23a	General interpretation	Discussion.
23b	Limitations of evidence	Strengths and Limitations.
23c	Limitations of review process	Strengths and Limitations.
23d	Implications	Implications for Clinical Practice and Future Research.
24a	Registration	Methods: review not registered.
24b	Protocol access	Methods: no public protocol.
24c	Protocol amendments	Not applicable.
25	Support	Funding statement.
26	Competing interests	Conflicts of Interest statement.
27	Availability of data, code, and materials	Data Availability statement and Multimedia Appendices A-D.
