# Supplementary material for: AI-Driven and Automated Systems for Continuous Oxygen Saturation Monitoring in Long-Term Oxygen Therapy: Systematic Review
Source: JMIRx Med. 2026 Oct 7;7:e76506. doi: 10.2196/76506 (PMC13646447; doi:10.2196/76506)
Supplement: Supplementary file 2 [file xmed-v7-e76506-s001.docx]

**Appendix 1: Inclusion and exclusion Criteria, search Strategies, and data Extraction Form**

This appendix provides the source-specific search strategies, eligibility criteria, screening counts, inclusion-hit reconciliation, and data extraction fields used for the revised systematic review. The reconciliation table distinguishes source-level inclusion hits before cross-source deduplication from the final set of 8 unique included studies.

# Eligibility Criteria

| Domain | Inclusion criteria | Exclusion criteria |
| --- | --- | --- |
| Population and setting | Adults or LTOT-relevant clinical, home, simulated, or mixed cohorts involving oxygen therapy, oxygen titration, oxygen saturation monitoring, COPD, chronic respiratory disease, or chronic oxygen use. | Acute surgical or procedural oxygen-only studies with no relevance to LTOT, home oxygen, COPD or chronic respiratory disease, or sustained oxygen titration. |
| Technology | AI, machine learning, predictive modeling, automation, closed-loop control, or smart-device approaches linked to SpO2 monitoring or oxygen delivery. | Studies without AI, machine learning, automation, or closed-loop oxygen-control relevance. |
| Measurement focus | SpO2, pulse oximetry, photoplethysmography, oxygen saturation prediction, oxygen-flow titration, time in target range, hypoxemia, or hyperoxemia outcomes. | Studies where SpO2 or oxygen delivery was not a primary or directly relevant outcome. |
| Signal-quality relevance | Studies addressing or reporting low perfusion, motion artifact, skin tone or pigmentation, measurement reliability, long-term stability, or validation constraints were eligible when otherwise relevant. | Papers limited to unrelated sensor engineering without LTOT, SpO2, or oxygen-delivery relevance. |
| Publication type and language | Peer-reviewed journal articles and peer-reviewed conference proceedings where applicable, open access or accessible full text, English language, 2000 to January 2025. | Non-English reports, editorials, theses, conference abstracts without full papers, unavailable full texts, and paywalled records that could not be assessed. |

# PRISMA-Level Record Flow

| Record flow element | Count |
| --- | --- |
| Database and platform records identified | 926 |
| Manual reference records identified | 2 |
| Total records before duplicate removal | 928 |
| Duplicates removed | 16 |
| Records screened | 912 |
| Full-text articles assessed for eligibility after merged screening | 61 |
| Full-text articles excluded | 53 |
| Unique studies included in qualitative synthesis | 8 |

# Source-Level Inclusion-Hit Reconciliation

Per-source included counts are reported here as inclusion hits before final cross-source study deduplication. These hits do not equal the final number of unique included studies until overlap between sources is removed.

| Source | Records identified | Source-level screening record | Inclusion hits before study-level reconciliation | Final unique contribution |
| --- | --- | --- | --- | --- |
| PubMed | 111 | All 111 records were screened by title and abstract; 15 records were selected for full-text review in the source log. | 4 hits: Pascual-Saldana et al. (2024); Sanchez-Morillo et al. (2020); Hansen et al. (2018); Cirio and Nava (2011). | 4 unique studies. |
| IEEE Xplore | 23 | 3 records were selected after title and abstract screening. | 1 hit: Pascual et al. (2023). | 1 unique study. |
| Springer Link | 50 | 5 full-text records were assessed in the source log. | 0 hits. | 0 unique studies. |
| MDPI supplementary searches | 11 | Five targeted MDPI searches were performed; 3 inclusion hits were recorded in the source log. | 3 hits: Sanchez-Morillo et al. (2020); Pascual-Saldana et al. (2024); Cabanas et al. (2024). | 1 unique study after removing 2 PubMed-overlap hits: Cabanas et al. (2024). |
| ACM Digital Library | 731 | 15 records were selected after title and abstract screening; 5 full texts were assessed where accessible. | 0 hits. | 0 unique studies. |
| Manual reference screening | 2 | Backward/reference-list screening identified 2 additional records. | 2 hits: Shuzan et al. (2023); Arguello-Prada and Castillo Garcia (2024). | 2 unique studies. |
| Total reconciliation | 928 total records before duplicate removal | After 16 record duplicates were removed, 912 records were screened and 61 full texts were assessed after merged screening. | 10 source-level inclusion hits: PubMed 4 + IEEE 1 + MDPI 3 + manual 2. | 8 unique included studies after removing 2 MDPI/PubMed overlap hits. |

## Explicit Overlap Reconciliation

The source-level inclusion hits summed to 10 before study-level reconciliation: PubMed 4 + IEEE Xplore 1 + MDPI supplementary searches 3 + manual reference screening 2. Two MDPI inclusion hits were duplicate study-level hits already identified in PubMed: Sanchez-Morillo et al. (2020) and Pascual-Saldana et al. (2024). After removing these two overlapping inclusion hits, the final included set was 8 unique studies: 4 PubMed unique contributions, 1 IEEE Xplore unique contribution, 1 MDPI unique contribution, and 2 manual-reference unique contributions.

# Included Studies After Reconciliation

| Manuscript ref | Study | Correct title | Technology category | Source attribution after reconciliation |
| --- | --- | --- | --- | --- |
| 5 | Shuzan et al. (2023) | Machine Learning-Based Respiration Rate and Blood Oxygen Saturation Estimation Using Photoplethysmogram Signals | AI-based SpO2 and respiratory-rate estimation from PPG signals. | Manual reference screening; unique included study. |
| 6 | Cabanas et al. (2024) | Evaluating AI Methods for Pulse Oximetry: Performance, Clinical Accuracy, and Comprehensive Bias Analysis | AI pulse-oximetry performance, clinical accuracy, and bias analysis. | MDPI supplementary search; unique MDPI contribution. |
| 7 | Sanchez-Morillo et al. (2020) | Automated Home Oxygen Delivery for Patients with COPD and Respiratory Failure: A New Approach | Automated non-AI home oxygen delivery for COPD and respiratory failure. | PubMed inclusion hit; duplicate inclusion hit also appeared in MDPI and was removed during study-level reconciliation. |
| 8 | Hansen et al. (2018) | Automated oxygen control with O2matic during admission with exacerbation of COPD | Automated non-AI closed-loop oxygen control during COPD exacerbation admission. | PubMed inclusion hit; unique included study. |
| 9 | Arguello-Prada and Castillo Garcia (2024) | Machine Learning Applied to Reference Signal-Less Detection of Motion Artifacts in Photoplethysmographic Signals: A Review | Machine-learning signal-quality and motion-artifact review relevant to PPG and SpO2 reliability. | Manual reference screening; unique included study. |
| 10 | Pascual-Saldana et al. (2024) | Innovative Predictive Approach towards a Personalized Oxygen Dosing System | AI and edge-computing framework for personalized oxygen dosing. | PubMed inclusion hit; duplicate inclusion hit also appeared in MDPI and was removed during study-level reconciliation. |
| 13 | Pascual et al. (2023) | Analyzing distinct neural network models for oxygen saturation prediction towards a personalized COPD management | IEEE conference study comparing neural-network models for SpO2 prediction. | IEEE Xplore inclusion hit; unique included study. |
| 14 | Cirio and Nava (2011) | Pilot study of a new device to titrate oxygen flow in hypoxic patients on long-term oxygen therapy | Automated non-AI oxygen-flow titration in hypoxic LTOT patients. | PubMed inclusion hit; unique included study. |

# Source-Specific Search Strategies

| Source | Search query and limits | Screening and inclusion notes |
| --- | --- | --- |
| PubMed | Query: (("oxygen therapy" OR "long-term oxygen therapy" OR LTOT OR "oxygen titration" OR "oxygen delivery" OR "oxygen therapy") AND ("artificial intelligence" OR "machine learning" OR "deep learning" OR "automated system" OR "closed-loop" OR automation OR "smart device" OR "predictive model" OR "digital health" OR "closed-loop") AND ("SpO2" OR "oxygen saturation" OR "pulse oximetry" OR photoplethysmography OR PPG OR oximeter)). Limits: English, 2000 to January 2025. | Records identified: 111. Source-level full texts selected: 15. Inclusion hits: Pascual-Saldana et al. (2024), Sanchez-Morillo et al. (2020), Hansen et al. (2018), and Cirio and Nava (2011). Final unique contribution: 4. |
| IEEE Xplore | Same core concepts: oxygen therapy or LTOT or oxygen titration or oxygen delivery; artificial intelligence, machine learning, deep learning, automation, closed-loop, smart device, predictive model, or digital health; and SpO2, oxygen saturation, pulse oximetry, photoplethysmography, PPG, or oximeter. Limits: English, 2000 to January 2025. | Records identified: 23. Title/abstract-selected records: 3. Inclusion hit and final unique contribution: Pascual et al. (2023). |
| Springer Link | Query: (("chronic obstructive pulmonary disease" OR COPD OR "chronic lung disease") AND ("digital health" OR "eHealth" OR "remote monitoring" OR "telemedicine") AND ("artificial intelligence" OR "machine learning" OR "deep learning" OR "predictive model" OR "automated system" OR "closed-loop" OR automation OR "smart device") AND ("oxygen therapy" OR "long-term oxygen therapy" OR LTOT OR "oxygen titration" OR "oxygen delivery") AND ("SpO2" OR "oxygen saturation" OR "pulse oximetry" OR photoplethysmography OR PPG OR oximeter)). Limits: English, open access, 2000 to January 2025. | Records identified: 50. Full texts assessed in source log: 5. Inclusion hits: 0. Final unique contribution: 0. |
| MDPI supplementary searches | Searches: "Oxygen Delivery" AND "LTOT" AND "Automated"; "Oxygen Delivery" AND "LTOT" AND "Artificial Intelligence"; "Oxygen Delivery" AND "LTOT" AND "Machine Learning"; "COPD" AND "LTOT" AND "Machine Learning"; "SpO2" AND ("Artificial Intelligence" OR "Machine Learning"). Limits: English, open access, 2000 to January 2025. | Records identified: 11. Inclusion hits: Sanchez-Morillo et al. (2020), Pascual-Saldana et al. (2024), and Cabanas et al. (2024). Sanchez-Morillo et al. and Pascual-Saldana et al. overlapped with PubMed hits; final unique MDPI contribution: Cabanas et al. (2024). |
| ACM Digital Library | Query: ("Machine Learning" OR "Artificial Intelligence") AND (SpO2 OR "oxygen delivery"). Limits: English, 2000 to January 2025, full text assessed where accessible. | Records identified: 731. Title/abstract-selected records: 15. Full texts assessed in source log: 5. Inclusion hits: 0. Final unique contribution: 0. |
| Manual reference screening | Backward and reference-list screening of included and closely related articles. | Records identified: 2. Inclusion hits and final unique contributions: Shuzan et al. (2023) and Arguello-Prada and Castillo Garcia (2024). |

## ACM Digital Library Records Selected for Full-Text Review

| Record | Disposition |
| --- | --- |
| A Machine-Learning-Based Prediction Method for Easy COPD Classification Based on Pulse Oximetry | Excluded: classification focus rather than LTOT oxygen titration. |
| Smartphone-Based SpO2 Measurement Using Near-IR and Chromophore Compensation | Excluded: SpO2 measurement engineering without LTOT oxygen titration. |
| Screening of Finger Pulse Oximeter Pre-Calibration Function Test with Machine Learning | Excluded: device calibration focus without LTOT application. |
| Deep Learning Approach to Estimate SpO2 from PPG Signals | Excluded: signal-estimation focus without LTOT oxygen delivery. |
| Automated Medical Oxygen Regulator Using Blood Oxygen Saturation Level | Excluded: insufficient LTOT-specific evidence or inaccessible full assessment details. |

# Data Extraction Form

| Field | Extraction instruction |
| --- | --- |
| Citation | First author, year, title, journal or proceedings, DOI if available. |
| Country and setting | Country, clinical or home setting, simulated/laboratory setting, inpatient setting, or mixed setting. |
| Study design | Prototype validation, observational analysis, randomized or nonrandomized trial, pilot study, review, or simulation. |
| Population | Condition, LTOT status or oxygen-dependence relevance, sample size, and inclusion/exclusion criteria. |
| Technology type | AI, machine learning, predictive model, automation, closed-loop controller, smart oxygen-delivery device, or pulse-oximetry method. |
| Algorithm or device | Named model, control system, device, sensors, SpO2 input, oxygen-flow output, and intervention logic. |
| Outcomes | SpO2 prediction accuracy, time in target saturation range, oxygen-flow adjustment, hypoxemia, hyperoxemia, safety, usability, or feasibility. |
| Validation and comparator | Internal/external validation, clinical comparator, manual titration comparator, usual care, or no comparator. |
| Equity and signal quality | Any findings on skin tone, perfusion, motion artifact, sensor reliability, or long-term signal stability. |
| Risk of bias | D1 to D7 domain ratings and overall narrative rating. |
| Reviewer notes | Reviewer initials, conflicts, reconciliation notes, and reasons for exclusion if applicable. |
