# Supplementary material for: AI-Driven and Automated Systems for Continuous Oxygen Saturation Monitoring in Long-Term Oxygen Therapy: Systematic Review
Source: JMIRx Med. 2026 Oct 7;7:e76506. doi: 10.2196/76506 (PMC13646447; doi:10.2196/76506)
Supplement: Supplementary file 3 [file xmed-v7-e76506-s002.docx]

**Multimedia appendix 2: Risk of bias materials**

This appendix provides the risk-of-bias domain definitions, study-level judgements, and reproducible coding structure used to support the revised manuscript. The overall manuscript rating is narrative and reflects the synthesis reported in Table 2 of the main manuscript.

# Domain Definitions

| Domain | Definition |
| --- | --- |
| D1 | Bias due to confounding. |
| D2 | Bias due to selection of participants. |
| D3 | Bias in classification of interventions. |
| D4 | Bias due to deviations from intended interventions. |
| D5 | Bias due to missing data. |
| D6 | Bias in measurement of outcomes. |
| D7 | Bias in selection of the reported result. |

# Study-Level Ratings

| Study | D1 | D2 | D3 | D4 | D5 | D6 | D7 | Overall manuscript rating |
| --- | --- | --- | --- | --- | --- | --- | --- | --- |
| Cabanas et al. (2024) | Moderate | Moderate | Low | Low | Low | Low | Low | Low-Moderate |
| Shuzan et al. (2023) | Moderate | Serious | Low | Moderate | Moderate | Moderate | Moderate | Moderate-Serious |
| Arguello-Prada and Castillo (2024) | Moderate | Serious | Moderate | Moderate | Moderate | Moderate | Moderate | Moderate-Serious |
| Pascual-Saldana et al. (2024) | Moderate | Moderate | Low | Moderate | Moderate | Moderate | Moderate | Moderate |
| Pascual et al. (2023) | Serious | Moderate | Serious | Serious | Moderate | Serious | Serious | Serious |
| Sanchez-Morillo et al. (2020) | Moderate | Moderate | Low | Low | Low | Moderate | Moderate | Moderate |
| Hansen et al. (2018) | Moderate | Moderate | Low | Low | Low | Moderate | Moderate | Low-Moderate |
| Cirio and Nava (2011) | Serious | Moderate | Low | Low | Low | Serious | Serious | Serious |

# Risk of Bias Figure

The figure below is a grayscale rendering of the standard robvis output. The table above contains the manuscript narrative overall ratings. The plotting code uses the nearest standard ROBINS-I overall category because robvis accepts standard categorical levels.

# R Code Structure

library(robvis)
library(ggplot2)

risk_of_bias_mod <- data.frame(
 Study = c("Cabanas et al. (2024)", "Shuzan et al. (2023)",
 "Arguello-Prada and Castillo (2024)", "Pascual-Saldana et al. (2024)",
 "Pascual et al. (2023)", "Sanchez-Morillo et al. (2020)",
 "Hansen et al. (2018)", "Cirio and Nava (2011)"),
 Domain1 = c("Moderate", "Moderate", "Moderate", "Moderate", "Serious", "Moderate", "Moderate", "Serious"),
 Domain2 = c("Moderate", "Serious", "Serious", "Moderate", "Moderate", "Moderate", "Moderate", "Moderate"),
 Domain3 = c("Low", "Low", "Moderate", "Low", "Serious", "Low", "Low", "Low"),
 Domain4 = c("Low", "Moderate", "Moderate", "Moderate", "Serious", "Low", "Low", "Low"),
 Domain5 = c("Low", "Moderate", "Moderate", "Moderate", "Moderate", "Low", "Low", "Low"),
 Domain6 = c("Low", "Moderate", "Moderate", "Moderate", "Serious", "Moderate", "Moderate", "Serious"),
 Domain7 = c("Low", "Moderate", "Moderate", "Moderate", "Serious", "Moderate", "Moderate", "Serious"),
 Overall = c("Moderate", "Serious", "Serious", "Moderate", "Serious", "Moderate", "Moderate", "Serious"),
 Overall_narrative = c("Low-Moderate", "Moderate-Serious", "Moderate-Serious", "Moderate", "Serious", "Moderate", "Low-Moderate", "Serious")
)

rob_summary(data = risk_of_bias_mod[, c("Study", "Domain1", "Domain2", "Domain3", "Domain4", "Domain5", "Domain6", "Domain7", "Overall")], tool = "ROBINS-I")
rob_traffic_light(data = risk_of_bias_mod[, c("Study", "Domain1", "Domain2", "Domain3", "Domain4", "Domain5", "Domain6", "Domain7", "Overall")], tool = "ROBINS-I")
