# Supplementary material for: AI-Driven and Automated Systems for Continuous Oxygen Saturation Monitoring in Long-Term Oxygen Therapy: Systematic Review
Source: JMIRx Med. 2026 Oct 7;7:e76506. doi: 10.2196/76506 (PMC13646447; doi:10.2196/76506)
Supplement: Supplementary file 4 [file xmed-v7-e76506-s004.docx]

**PRISMA-S checklist**

Checklist source: Rethlefsen ML et al. PRISMA-S: an extension to the PRISMA Statement for Reporting Literature Searches in Systematic Reviews. Systematic Reviews. 2021;10:39. This table summarizes where search-reporting elements are addressed in the revised manuscript and appendices.

| Item | Topic | Status or location in revised submission |
| --- | --- | --- |
| 1 | Database name | Methods: Information Sources and Search Strategy; Appendix A source-level search tables. |
| 2 | Multi-database searching | Searches were conducted separately by source or platform; counts are reconciled in Appendix A and Figure 1. |
| 3 | Study registries | Not searched; not applicable to the review scope. |
| 4 | Online resources and browsing | Springer Link and MDPI publisher/platform searches are reported in Appendix A. |
| 5 | Citation searching | Manual reference screening identified 2 records; reported in Figure 1, Results, and Appendix A. |
| 6 | Contacts | No author or investigator contact was used for study identification. |
| 7 | Other methods | Manual reference screening is reported in Appendix A. |
| 8 | Full search strategies | Full source-specific search strings and limits are reported in Appendix A. |
| 9 | Limits and restrictions | English language, accessible full text or open access where required, and 2000 to January 2025 are reported in Methods and Appendix A. |
| 10 | Search filters | Disease, intervention, AI/automation, and SpO2 concepts are documented in Appendix A. |
| 11 | Prior work | Search concepts were based on LTOT, automation or AI, SpO2 monitoring, oxygen titration, and signal-quality themes described in the Introduction and Methods. |
| 12 | Search updates | Searches were completed in January 2025; no formal update search is reported. |
| 13 | Dates of searches | The search window and January 2025 final search timing are reported in Methods and Appendix A. |
| 14 | Peer review | No formal PRESS peer review is reported; two-reviewer screening and reconciliation are described in Methods. |
| 15 | Total records | Records identified, screened, full texts assessed, and included studies are reported in Results, Figure 1, and Appendix A. |
| 16 | Deduplication | Sixteen duplicates were removed; reported in Figure 1 and Appendix A. |
